# The causal relationship between immune cell traits and schizophrenia: a Mendelian randomization analysis

**DOI:** 10.3389/fimmu.2024.1452214

**Published:** 2024-09-27

**Authors:** Jianbin Du, Ancha Baranova, Guofu Zhang, Fuquan Zhang

**Affiliations:** ^1^ Department of Geriatric Psychiatry, The Affiliated Mental Health Center of Jiangnan University, Wuxi Central Rehabilitation Hospital, Wuxi, Jiangsu, China; ^2^ School of Systems Biology, George Mason University, Fairfax, VA, United States; ^3^ Research Centre for Medical Genetics, Moscow, Russia; ^4^ Department of Psychiatry, The Affiliated Brain Hospital of Nanjing Medical University, Nanjing, China; ^5^ Institute of Neuropsychiatry, The Affiliated Brain Hospital of Nanjing Medical University, Nanjing, China

**Keywords:** schizophrenia, immune cell, immunology, psychiatry, Mendelian randomization analysis

## Abstract

**Introduction:**

The complex and unresolved pathogenesis of schizophrenia has posed significant challenges to its diagnosis and treatment. While recent research has established a clear association between immune function and schizophrenia, the causal relationship between the two remains elusive.

**Methods:**

We employed a bidirectional two-sample Mendelian randomization approach to investigate the causal relationship between schizophrenia and 731 immune cell traits by utilizing public GWAS data. We further validated the causal relationship between schizophrenia and six types of white cell measures.

**Results:**

We found the overall causal effects of schizophrenia on immune cell traits were significantly higher than the reverse ones (0.011 ± 0.049 vs 0.001 ± 0.016, p < 0.001), implying that disease may lead to an increase in immune cells by itself. We also identified four immune cell traits that may increase the risk of schizophrenia: CD11c+ monocyte %monocyte (odds ratio (OR): 1.06, 95% confidence interval (CI): 1.03~1.09, FDR = 0.027), CD11c+ CD62L- monocyte %monocyte (OR:1.06, 95% CI: 1.03~1.09, FDR = 0.027), CD25 on IgD+ CD38- naive B cell (OR:1.03, 95% CI:1.01~1.06, FDR = 0.042), and CD86 on monocyte (OR = 1.04, 95% CI:1.01~1.06, FDR = 0.042). However, we did not detect any significant causal effects of schizophrenia on immune cell traits. Using the white blood cell traits data, we identified that schizophrenia increases the lymphocyte counts (OR:1.03, 95%CI: 1.01-1.04, FDR = 0.007), total white blood cell counts (OR:1.02, 95%CI: 1.01-1.04, FDR = 0.021) and monocyte counts (OR:1.02, 95%CI: 1.00-1.03, FDR = 0.034). The lymphocyte counts were nominally associated with the risk of schizophrenia (OR:1.08,95%CI:1.01-1.16, P=0.019).

**Discussion:**

Our study found that the causal relationship between schizophrenia and the immune system is complex, enhancing our understanding of the role of immune regulation in the development of this disorder. These findings offer new insights for exploring diagnostic and therapeutic options for schizophrenia.

## Introduction

Schizophrenia is a severe mental disorder characterized by psychosis as a hallmark symptomatic manifestation (1). Approximately 1% of the global population will experience schizophrenia in their lifetime (2), and about 5% of these patients face increased physical health problems and elevated suicide rates (2, 3). The higher morbidity and disability rates associated with schizophrenia result in an average life expectancy that is 10-25 years shorter than that of the general population (4, 5), representing a profound loss for families and a substantial health burden on society (6). The complex etiology of schizophrenia complicates early detection and timely diagnosis, while the diversity of symptoms and conditions challenges the effective implementation of treatment strategies (7, 8). To achieve individualized and precise medical treatment for schizophrenia and facilitate the reintegration of patients into society, it is essential to explore the mechanisms of action and identify biomarkers related to its pathogenesis.

Inflammatory factors have a quantitative impact on brain development, typically aiding in processes such as neurogenesis, neuronal migration, and synapse formation (9–12). However, excessive inflammatory responses can disrupt these processes (13), and the release of large amounts of inflammatory factors in the brain increases the risk of psychiatric disorders, including epilepsy (14), Alzheimer’s disease (15), depression (16), and schizophrenia (17). Epidemiologic studies have long established an association between early-life infections and the development of autoimmune diseases and subsequent psychiatric disorders in adulthood (18). Numerous studies on chronic and first-episode schizophrenia have shown elevated levels of interleukin (IL)-1β, IL-6, IL-8, tumor necrosis factor (TNF), and C-reactive proteins in peripheral blood and cerebrospinal fluid (19–22), suggesting that neuroinflammation is a key pathophysiological mechanism of schizophrenia (21, 23). Genome-wide association studies (GWAS) have corroborated these findings, indicating a close relationship between genes regulating immune responses and schizophrenia (24, 25). Therefore, investigating the interactions between immune function and psychiatric disorders is crucial for understanding the pathogenesis of these conditions and may provide valuable insights for their treatment. Nevertheless, current findings on the association between immune inflammation and schizophrenia remain inconsistent, likely due to limitations in sample sizes, study design flaws, and uncontrolled confounding variables (26–28).

To mitigate confounding factors and reduce the effects of reverse causality commonly encountered in cross-sectional studies, we employed Mendelian randomization (MR) to analyze data from a GWAS of schizophrenia and two cell traits. This approach was widely used in recent studies on somatic (29–31) and mental disorders (32–37). We aim to uncover potential causal relationships between schizophrenia and immune function, thereby identifying potential biomarkers and facilitating individualized and precise treatment for schizophrenia.

## Materials and methods

### Overall study design

Using the GWAS public database, we conducted a bidirectional two-sample MR study. In the forward MR analysis, cell traits were used as exposures and schizophrenia as the outcome, and in the reverse MR analysis, schizophrenia was used as the exposure and cell traits as outcomes. The selection of instrumental variables adhered to the three core principles of MR research: 1) the genetic variant must be directly associated with the exposure; 2) the genetic variant should be independent of confounders related to the exposure or outcomes; and 3) the genetic variant must not influence the outcome except through its association with the exposure. Ethical review and informed consent were obtained for all GWAS study cohorts.

### Data source of schizophrenia and two cell traits

The GWAS summary data on schizophrenia were acquired from the Psychiatric Genomics Consortium (PGC), including 53,386 cases and 77,258 controls (38). GWAS summary data for immune cell traits were collected from a cohort of 3,757 Sardinians by a team of Italian researchers, aiming to elucidate immune system function and dysfunction by assessing the impact of natural genetic variation on both quantitative and discrete immune-related traits (39). This study analyzed 731 immune cell traits, categorized into 4 types: absolute counts (AC, n=118), morphometric parameters (MP, n=32), median fluorescence intensity (MFI, n=389) analyzed by flow cytometry, and relative counts (RC, n=192) calculated using ratios between cell levels. The MFI, AC, and RC types included B cells, circulating dendritic cells (cDC), mature stage T cells, monocytes, myeloid cells, TBNK (T cells, B cells, natural killer cells), and Treg panels. The MP type encompassed only cDC and TBNK panels. This study cohort was adjusted for gender and age covariates, and its summary-level statistics are publicly available in the GWAS catalog (https://www.ebi.ac.uk/gwas/studies/) under accession numbers GCST0001391 to GCST0002121. GWAS summary data for six white blood cell traits were acquired from a large study which assessed the relevance of the omnigenic model to blood cell phenotypes in 563,085 participants (40).

### Selection of instrumental variables

We selected single nucleotide polymorphisms (SNPs) with large exposure-related effect sizes and high statistical significance as reliable instrumental variables (IVs) based on the principles of MR analysis. To avoid biased parameter estimates due to low statistical power and weak IVs during MR analysis, we applied different significance thresholds for associations under varying exposure conditions. For immune cell traits used as exposures, a significance level of 1 × 10⁻⁵ was employed for extracting IVs for each trait, in line with a precedent set in a prior study (41). For schizophrenia and blood cell traits used as exposures for IV extraction, a more stringent significance level of 5 × 10⁻⁸ was maintained. Independent SNPs were selected through linkage disequilibrium clumping (r² threshold < 0.001 within a 10,000 kb distance) based on the European 1000 Genomes reference panel. To satisfy the first assumption of MR and to estimate weak instrument bias and instrument strength, we calculated F-statistics (β²/SE²), recommending F-statistics > 10 for further MR analysis. Ultimately, we identified 3 to 640 independent IVs across various immune cell traits, along with 194 to 587 IVs in different blood cell traits and 147 IVs related to schizophrenia for subsequent analysis.

### Statistical methods

We use the random-effects IVW method to assess the potential bidirectional causality between immune cell traits and schizophrenia, which guarantees robust causal estimates even in the presence of heterogeneity in genetic instruments (42). Other methods serve as complement methods to IVW like MR-Egger, which can enhance the reliability and validity of causal inferences in MR studies by addressing the potential confounding effects of pleiotropy (43). The weighted median also was used to provide ancillary support for the reliability of the causal results. Additionally, we conducted sensitivity analyses to evaluate pleiotropy and heterogeneity, aiming to avoid violating the second or third assumptions of MR. The MR-Egger intercept method was used to determine the presence of horizontal pleiotropy in the genetic instruments, and pleiotropy was considered to be absent when P > 0.05. Heterogeneity was tested using Cochran’s Q test, which indicated the existence of heterogeneity when P<0.05. A sensitivity analysis was conducted to further determine the effect of heterogeneity through the leave-one-out method and to assess the robustness of the final effects. The MR analysis in this study utilized the TwoSampleMR (44) package based on the R language. All P values and false discovery rate (FDR) are two-tailed in this study.

## Results

### Comparison of overall causal effect across different exposure conditions

**Figure 1:**
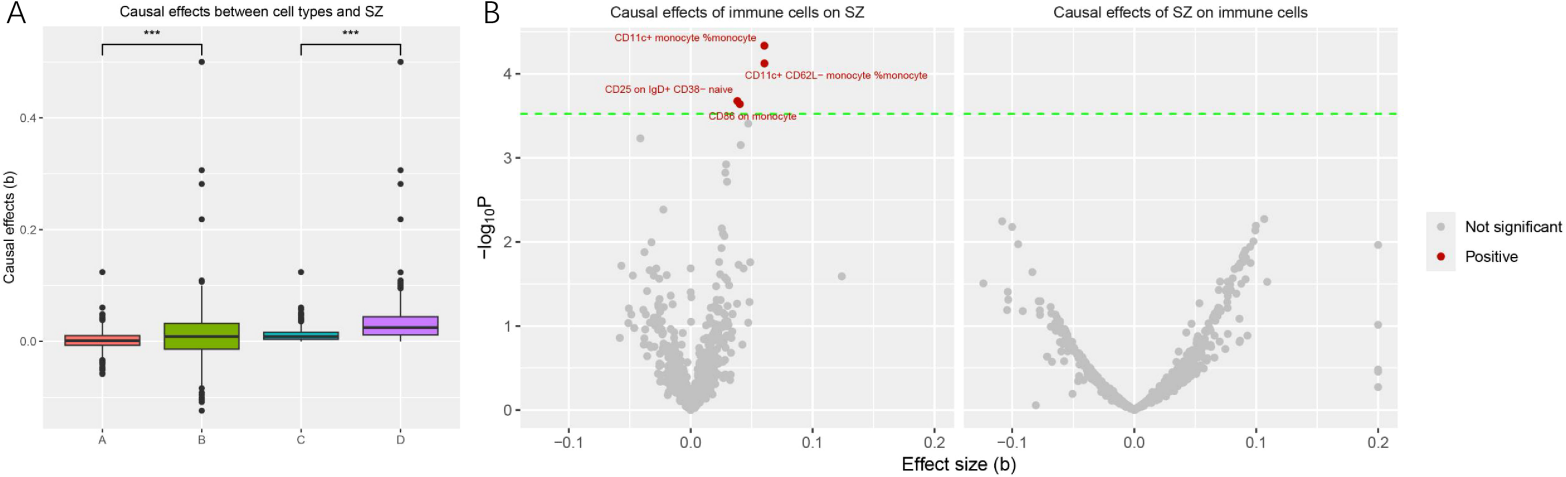
Comparison of causal effects size under different exposures and significant immune cell traits significantly associated with schizophrenia onset. **(A)** Each point in the box plots represents the causal effect of each exposure on the outcome. The horizontal coordinate A, B, C, and D represent the effect of cell traits on schizophrenia, the effect of schizophrenia on cell traits, the absolute effect of cell traits on schizophrenia, and the absolute effect of schizophrenia on cell traits, respectively. The vertical coordinate represents the magnitude of the causal effect size (beta value). (***, P < 0.001) **(B)** The volcano plot revealed four immune cell traits that were significantly and causally associated with the onset of schizophrenia. Significant cell traits are colored (red for positive relationships with schizophrenia, gray for not significant).

We compared the causal effect sizes under different exposure conditions first. The overall causal effect sizes (beta) of the genetic component predisposing to schizophrenia on immune cell traits were significantly higher than that of the reverse MR (beta exposure SZ = 0.011 ± 0.049, beta exposure cell traits = 0.001 ± 0.016, w = 308435, p < 0.001; |beta| exposure SZ = 0.033 ± 0.038, |beta| exposure cell traits = 0.002 ± 0.016, w = 461306, p < 0.001). A comparison of effect values for different exposure conditions is depicted in **Figure 1A**. We then specifically analyzed the causal relationship between schizophrenia and the two types of blood cells.

### Causal relationships between schizophrenia and 731 immune cell traits

**Figure 2:**
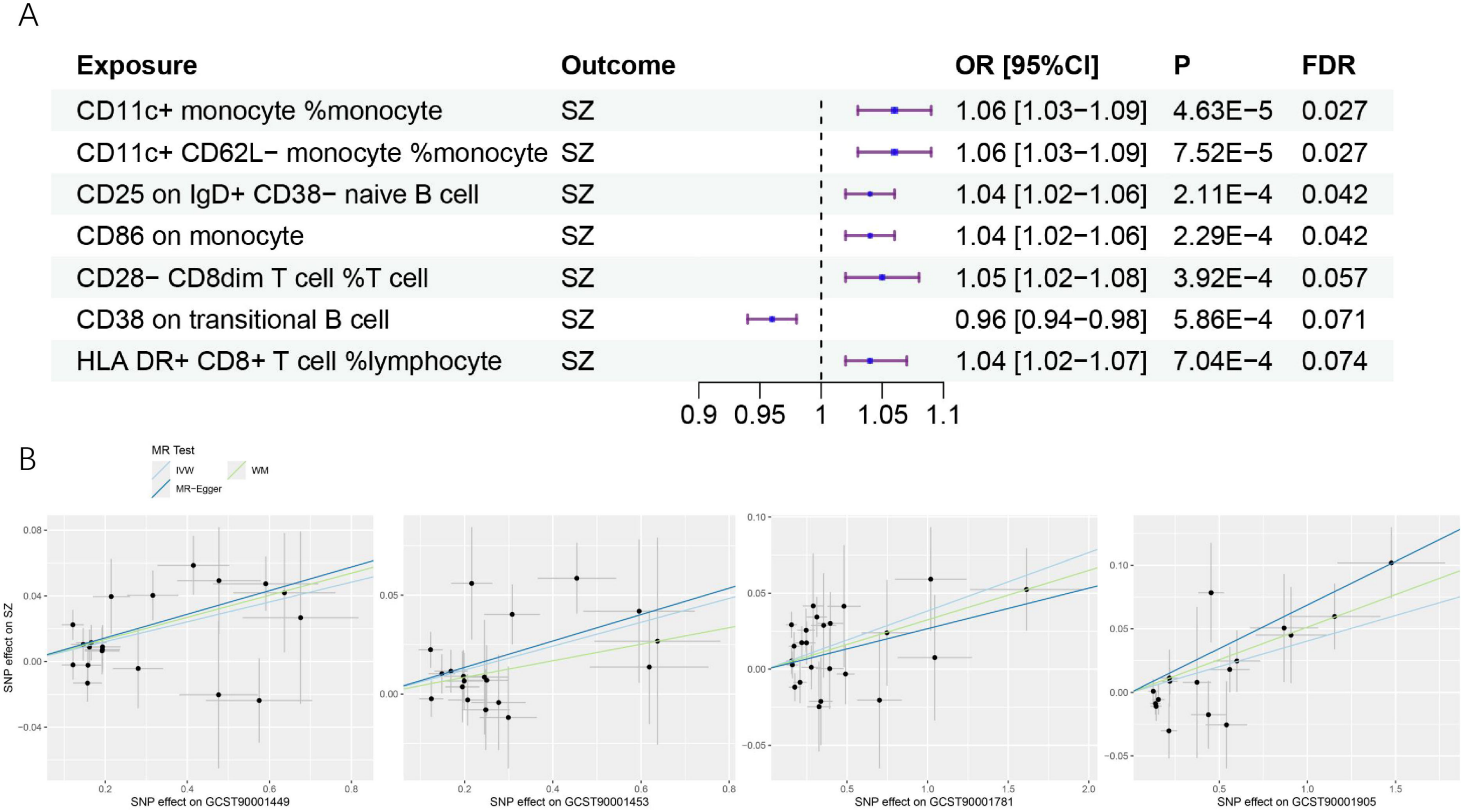
The causal effects of immune cell traits on schizophrenia. **(A)** Forest plots showed the causal associations of immune cell traits on schizophrenia. **(B)** Scatter plot showed the trends in the effect of CD11c+ monocyte %monocyte (trait ID: GCST90001449), CD11c+ CD62L- monocyte %monocyte (trait ID: GCST90001453), CD25 on IgD+ CD38- naive B cell (trait ID: GCST90001781), and CD86 on monocyte (trait ID: GCST90001905) on schizophrenia. SZ, schizophrenia; OR, Odds Ratio; CI, confidence interval; FDR, false discovery rate.

We found that a total of 4 immune cell traits were causally associated with SZ. They are CD11c+ monocyte %monocyte (odds ratio (OR): 1.06, 95% confidence interval (CI): 1.03~1.09, FDR = 0.027), CD11c+ CD62L- monocyte %monocyte (OR:1.06, 95% CI: 1.03~1.09, FDR = 0.027), CD25 on IgD+ CD38- naive B cell (OR:1.03, 95% CI:1.01~1.06, FDR = 0.042), and CD86 on monocyte (OR = 1.04, 95%CI:1.01~1.06, FDR = 0.042), respectively (**Table 1**, **Figure 2A**). All of them were identified as risk factors for schizophrenia onset (**Figure 1B**). Sensitive analysis results can be found in **Table 2** and **Figure 2B**. However, we did not find any causal effect of schizophrenia on immune cell traits (**Figure 1B**). Summary results of the causal relationship between schizophrenia and immune cell traits can be found in **Supplementary Tables 1** and **2**.

**Table 1:** Significant MR analysis results of immune cell traits on schizophrenia.

Exposure	Outcome	Method	N_IV	b (se)	OR [95%CI]	P	FDR
CD11c+ monocyte %monocyte	SZ	IVW	20	0.060 (0.015)	1.06 [1.03-1.09]	<0.001	0.027
		WM		0.067 (0.021)	1.07 [1.02-1.11]	0.001	
		MR-Egger		0.072 (0.029)	1.07 [1.01-1.13]	0.024	
CD11c+ CD62L- monocyte %monocyte	SZ	IVW	19	0.060 (0.015)	1.06 [1.03-1.09]	<0.001	0.027
		WM		0.042 (0.023)	1.04 [0.99-1.09]	0.064	
		MR-Egger		0.067 (0.034)	1.06 [0.99-1.14]	0.068	
CD25 on IgD+ CD38- naive B cell	SZ	IVW	24	0.038 (0.010)	1.03 [1.01-1.06]	<0.001	0.042
		WM		0.032 (0.015)	1.03 [1.00-1.06]	0.031	
		MR-Egger		0.027 (0.016)	1.02 [0.99-1.06]	0.12	
CD86 on monocyte	SZ	IVW	17	0.040 (0.011)	1.04 [1.01-1.06]	<0.001	0.042
		WM		0.051 (0.015)	1.05 [1.02-1.08]	0.001	
		MR-Egger		0.069 (0.014)	1.07 [1.04-1.10]	<0.001	

SZ, schizophrenia; IVW, inverse variance weighted; WM, weighted median; N_IV, number of instrumental variables; b, Beta; se, standard error; OR, odds ratio; CI, confidence interval.

**Table 2:** Sensitivity analysis of the effects of immune cell traits on schizophrenia.

			MR-Egger regression		Heterogeneity analyses		
Exposure	Outcome	Number of SNPs	Intercept	P	Method	Q	Q-pval
CD11c+ monocyte %monocyte	SZ	20			IVW	24.213	0.188
			-0.003	0.649	MR-Egger	23.929	0.157
CD11c+ CD62L- monocyte %monocyte	SZ	19			IVW	17.478	0.491
			-0.002	0.835	MR-Egger	17.432	0.426
CD25 on IgD+ CD38- naive B cell	SZ	24			IVW	28.081	0.213
			0.005	0.372	MR-Egger	27.06	0.209
CD86 on monocyte	SZ	17			IVW	18.123	0.317
			-0.015	0.014	MR-Egger	10.299	0.8

SZ, schizophrenia; SNP, single nucleotide polymorphism; MR, Mendelian randomization; IVW, inverse variance weighting.

### Causal relationships between schizophrenia and six white blood cell measures

**Figure 3:**
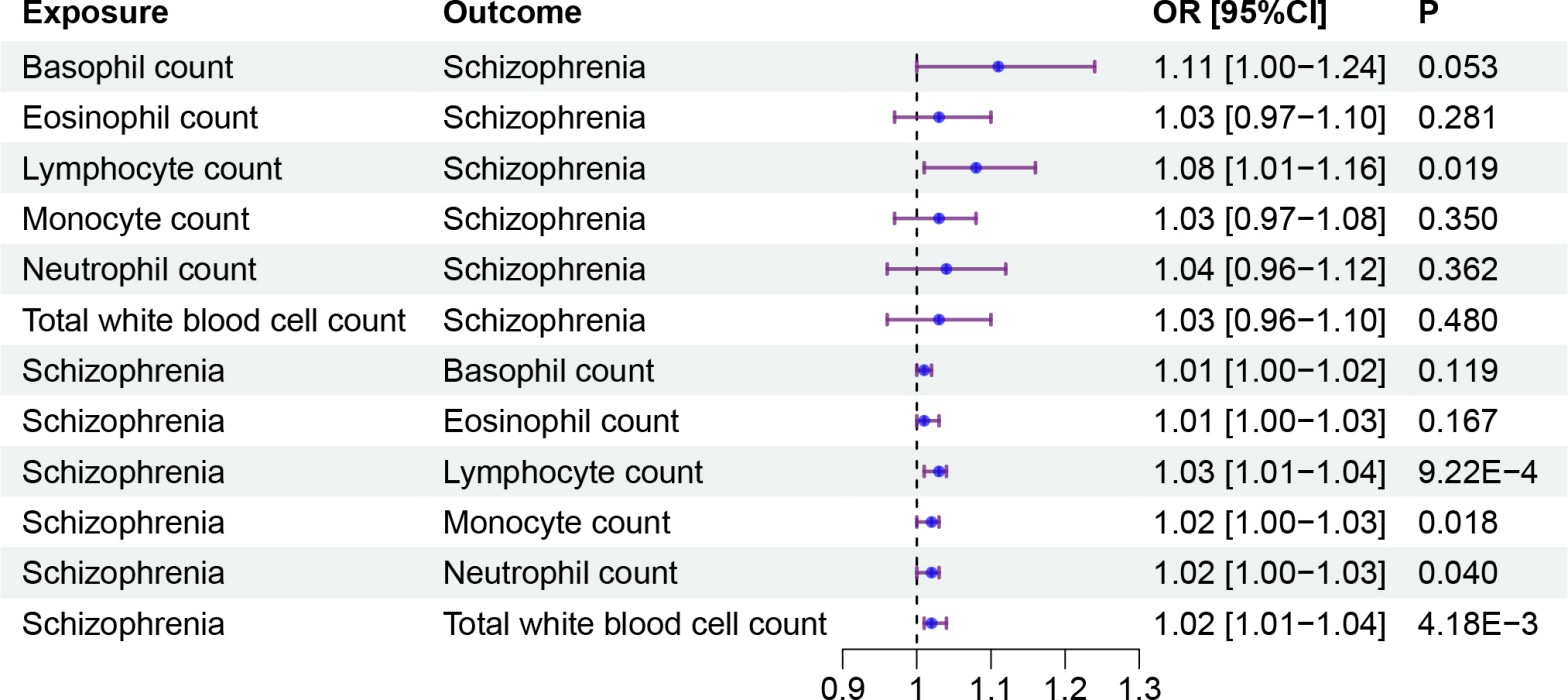
The causal effect between blood cell traits and schizophrenia. Forest plots showed the causal associations between blood cell traits and schizophrenia. OR, Odds Ratio; CI, confidence interval.

In a manner similar to described above, we found that the genetic predisposition to higher lymphocyte count had a positive causal effect on schizophrenia, although this effect was of nominal significance (OR: 1.08, 95%CI:1.01-1.16, P=0.019). We also found that the genetic signature predisposing to schizophrenia increased the level of three immune cell indicators, namely, lymphocyte count (OR: 1.03, 95%CI: 1.01-1.04, FDR = 0.007), total white blood cell count (OR: 1.02, 95%CI: 1.01-1.04, FDR = 0.021) and monocyte count (OR: 1.02, 95%CI: 1.00-1.03, FDR = 0.034) (**Figure 3**, **Table 3**). However, we did not find any causal effects of the genetic components defining blood cell related traits on schizophrenia. The results describing effects of blood cell traits on schizophrenia are presented in **Supplementary Tables 3**, **4**.

**Table 3:** Significant MR analysis results causally connecting schizophrenia and blood cell counts.

Exposure	Outcome	N_IV	b (se)	OR [95%CI]	P	FDR
Basophil count	SZ	194	0.105 (0.055)	1.11 [1.00-1.24]	0.053	0.269
Eosinophil count	SZ	427	0.033 (0.031)	1.03 [0.97-1.10]	0.281	0.602
Lymphocyte count	SZ	485	0.082 (0.035)	1.08 [1.01-1.16]	0.019	0.269
Monocyte count	SZ	505	0.026 (0.028)	1.03 [0.97-1.08]	0.35	0.603
Neutrophil count	SZ	416	0.035 (0.038)	1.04 [0.96-1.12]	0.362	0.603
Total white blood cell count	SZ	485	0.026 (0.036)	1.03 [0.96-1.10]	0.48	0.688
SZ	Basophil count	148	0.008 (0.005)	1.01 [1.00-1.02]	0.119	0.143
SZ	Eosinophil count	148	0.011 (0.008)	1.01 [1.00-1.03]	0.167	0.167
SZ	Lymphocyte count	148	0.027 (0.008)	1.03 [1.01-1.04]	9.22E-04	0.00553
SZ	Monocyte count	148	0.017 (0.007)	1.02 [1.00-1.03]	0.018	0.036
SZ	Neutrophil count	148	0.015 (0.007)	1.02 [1.00-1.03]	0.04	0.06
SZ	Total white blood cell count	148	0.024 (0.009)	1.02 [1.01-1.04]	4.18E-03	0.013

SZ, schizophrenia; N_IV, number of instrumental variables; b, Beta; se, standard error; OR, odds ratio; CI, confidence interval.

## Discussion

By analyzing the causal relationships between schizophrenia and blood cell traits, we identified a complex association between this disease and the characteristics of the immune system. Although the traits describing four immune cell types were identified as potential risk factors for the development of schizophrenia, the macroscopic perspective suggests that schizophrenia has a greater impact on immune cells than the reverse, and blood cell-related results have confirmed this finding.

Specifically, the genetic component predisposing to schizophrenia has resulted in increased white blood cell (WBC) counts, total lymphocyte counts, and total monocyte counts. These observations are consistent with the results of a recent MR study of the genetic links between schizophrenia and immune cells (45). In patients with schizophrenia, clinical studies have identified alterations in immune cell indicators. In particular, both in first-episode and in chronic schizophrenia patients, their neutrophil and monocyte counts are elevated at baseline, then, after medication-induced symptom improvement, they return to normal (46). Recent cross-sectional study also reported that patients with schizophrenia had elevated eosinophil, lymphocyte, monocyte, and neutrophil counts when compared to the normal population, and that both the neutrophil/lymphocyte ratio (NLR) and the monocyte/lymphocyte ratio (MLR) were significantly and positively correlated with the severity of negative symptoms (47). Two previous studies analyzed by META, although not agreeing on the consequences of antipsychotic drug interventions on immune cells, also detected the presence of elevated monocyte counts or lymphocytes in patients with schizophrenia (48, 49).

The underlying reasons behind this may be related to the involvement of the autonomic nervous system in the connection between the brain and the immune system (50, 51) and the activation of the immune system by psychosocial stresses (52, 53). Lymphoid organs innervated by sympathetic and peptidergic nerve fibers are able to promote certain immune responses under the action of neurotransmitters such as norepinephrine and substance P (54). On the other hand, lymphocytes, monocytes, and granulocytes possess neurotransmitter receptors, and are subjected to norepinephrine regulation (55, 56). In addition, it is well-known that social stress activates the HPA axis, increasing the levels of circulating glucocorticoids and leading to suppression of immune function (57). Schizophrenia, especially in its acute exacerbations, is associated with elevated levels of norepinephrine and an increase in chronic psychosocial stress, both of which are known to increase susceptibility to infections, which may be reflected in altered levels of immune cells.

It is important to note that the relationships between schizophrenia and the immune system are expected to be bidirectional. This phenomenon has been elucidated in previous studies (45, 58, 59). The important role that neuroinflammation plays in the development of schizophrenia is self-evident. In particular, the link between autoimmune disorders and schizophrenia identified earlier on suggests that these two conditions share a common underlying pathway (60, 61) of an inflammatory immune response. In addition to its own effects on the brain cells, inflammation is thought to increase the permeability of the blood-brain barrier and contribute to the penetration of the immune system components into the brain parenchyma (62). When neuroinflammation entrenches, microglia releases the cytokines that bind to specific receptors on the neurons and affect the release of the neurotransmitters, the synaptic plasticity, and the cortisol concentrations, leading to the changes in mood, the cognition, and the behavior (63, 64). It is, however, important to remember that the immune system is an intricate functional network involving many different cell population, which may play distinct roles in the pathogenesis of schizophrenia. Understanding the interrelationship connecting schizophrenia and immune cell traits may further improve the treatment strategies for this important neuropsychiatric disorder.

For example, monocytes, which are derived from hematopoietic stem cells embed in the bone marrow, are integral to the innate immune response, protecting the host from pathogen invasion and playing a crucial role in adaptive immunity (49). Elevated circulating monocyte levels have been observed in patients with schizophrenia, supported by the prevalence of mononucleosis in first-episode psychosis (65). A recent MR study further corroborates the pathogenic risk that monocytes pose to schizophrenia (45). Circulating monocytes are also suggested as indirect markers of microglia activation in the brain, with a noted correlation between increased monocyte levels and microglia activation (66, 67). It is proposed that microglia activation under pathological conditions may be part of the systemic activation of the mononuclear phagocyte system, leading to the activation of circulating monocytes (66, 68). The increased risk of schizophrenia associated with monocytes may be explained by the abnormal growth, differentiation, and dysfunction of neuronal circuits in the brain caused by early-life inflammatory activation (69), and/or the hyperactivation of microglia due to adverse environmental influences during adolescence (70).

B cells serve multiple functions in the adaptive immune system, including the production of immunoglobulins and the presentation of antigens to CD4+ T cells via the MHC-II complex (71). They also produce proinflammatory cytokines such as IL-6 and influence the recruitment of chemokines by monocytes and neutrophils (72, 73). IL-6 is particularly significant in B cell-related immune activation, with elevated levels often associated with schizophrenia (74). Autoreactive B cells also can produce autoantibodies, a process potentially triggered by environmental factors such as bacterial or viral infections, posing a risk factor for schizophrenia (60). Additionally, a distinct B cell subtype, B-1A cells, plays a crucial role in axonal myelin formation by stimulating oligodendrocyte production, which may also be implicated in the pathogenesis of schizophrenia (75).

Previous studies have cursorily explored the potential influence of immune cells on the pathophysiology of schizophrenia. The present study refines the understanding of the specific types of immune cells involved and establishes a causal relationship between immune cells and schizophrenia. These findings will inform future research on the interaction between immune function and the development of schizophrenia. However, this study has several limitations. First, the schizophrenia data cohort was derived from a European population, and caution is needed when applying the results to other populations. Second, the lack of demographic information prevented analysis of schizophrenia stratified by age and gender, limiting our understanding of how causality may vary across different age groups and sex. Third, the data describing the immune cell traits were derived from the general population of Sardinia and lacked specific information related to the status of general health in these individuals. Epidemiologic evidence suggests that due to known genetic drift, autoimmune diseases in this population are more common than in other Europeans populations (76). This should be considered when interpreting the conclusions of our study. In addition, we should not remain satisfied with current picture of the complex causal relationship between schizophrenia and immune cell traits, but endeavor on more in-depth studies such as exploring key genes and pathways linking the two, for example with the help of scPagwas methods (77) and scRNAseq data (78). Future gene- and pathway-level dissection should aid us in understanding the molecular mechanisms behind the uncovered causal relationships between the blood cell traits and the psychiatric diseases.

In conclusion, the causal relationship between schizophrenia and the immune system is complex, with the disease having a greater effect on immune cells, while the elevated risk of schizophrenia brought about by immune cells traits may be secondary. This finding enhances our understanding of the role of immune regulation in the development of this disorder and brings new insights for those exploring diagnostic and therapeutic options for schizophrenia.

## Data Availability

The original contributions presented in the study are included in the article/**Supplementary Material**. Further inquiries can be directed to the corresponding authors.
